# Research and young paediatricians

**DOI:** 10.1186/1824-7288-40-S1-A44

**Published:** 2014-08-11

**Authors:** M Loredana Marcovecchio

**Affiliations:** 1 Department of Paediatrics, University of Chieti, 66100, Italy

## 

The fundamental role played by medical research in the diagnosis and treatment of diseases is well recognized. In the paediatric field, research is crucial for the health of future generations. For a young paediatrician, being engaged in research requires curiosity and enthusiasm, strong commitment and constant dedication, but represents a unique opportunity to contribute to the progress of medical knowledge as well as to the overall progress of the society.

Education and training in research should be part of every level of the paediatric training, from premedical to postgraduate education. This will allow the development of research skills, which are useful not only for those who aim to pursue an academic career, but for the daily activity of every doctor. Research training can allow clinicians to keep up with the growing medical literature as well as improve their skills in finding the best evidence to answer questions arising from their daily clinical activity.

Over the last years, national policies have affected the role of scientific research and employment opportunities for young researchers. Italy has a number of PhD students lower than in other European countries. Furthermore, Italian PhD students’ salary, even after adjusting for the cost of living, is lower than that of their colleagues from Western European countries. In recent years, post-doctoral programs have also undergone considerable downsizing in terms of funds and availability.

Young people and their enthusiasm are essential for the advancement of research. Therefore, it is essential to promote and boost research in our country, through investments in scientific education of young doctors and by increasing the opportunities for those who wish to engage in medical research.

